# Effects of advanced glycation end products on neutrophil migration and aggregation in diabetic wounds

**DOI:** 10.18632/aging.202924

**Published:** 2021-04-26

**Authors:** Yutian Kang, Chongliang Zheng, Junna Ye, Fei Song, Xiqiao Wang, Yingkai Liu, Ming Tian, Jiaoyun Dong, Shuliang Lu

**Affiliations:** 1 Department of Burn, Shanghai Jiao Tong University Affiliated Ruijin Hospital, Shanghai, China; 2 Department of Rheumatology and Immunology, Shanghai Jiao Tong University Affiliated Ruijin Hospital, Shanghai, China

**Keywords:** diabetes, wound repair, neutrophil migration, AGEs, CTNND1

## Abstract

Increased accumulation of advanced glycation end products (AGEs) in diabetic skin is closely related to delayed wound healing. Studies have shown that the concentration of AGEs is elevated in the skin tissues and not subcutaneous tissues in refractory diabetic wounds, which suggests there may be a causal relationship between the two. In the present study, *in vitro* experiments revealed that AGEs activated neutrophils, and the migratory and adhesive functions of neutrophils decreased once AGE levels reached a certain threshold. Different levels of AGE expression differentially affected the function of neutrophils. Messenger RNA (mRNA) sequencing analysis combined with real-time polymerase chain reaction (PCR) showed that poliovirus receptor (PVR/CD155) and CTNND1, which play a role in migration- and adhesion-related signaling pathways, were decreased following AGE stimulation. Consequently, neutrophils cannot effectively stimulate the formation of the inflammatory belt needed to remove necrotic tissues and defend against foreign microorganisms within diabetic chronic wounds. In addition, this phenomenon may be related to the differential accumulation of AGEs in different layers of the skin.

## INTRODUCTION

Neutrophils are the first immune cells to infiltrate the wound area after skin injury. After neutrophils gather in the wound area, they form a dense inflammatory belt between the injured and normal tissues [1–3]. The normal functioning of neutrophils initiates the correct cellular signaling for the timely infiltration of macrophages [4, 5]. Dysregulation of neutrophilic-macrophagic stimulation may lead to an untimely, uncontrolled, and ineffective inflammatory reaction during the process of wound repair [6–8]. Previous studies revealed that there were significant differences in this process between diabetic and control conditions. Specifically, the formation of the inflammatory belt was delayed and neutrophil infiltration was increased in diabetic conditions as compared with those in control conditions [1, 9]. Additionally, Ge et al. [10] reversed these abnormalities in diabetic wounds using an advanced glycation end product (AGE) inhibitor, aminoguanidine (AG) [1]. Therefore, AGEs may contribute to these phenomena in diabetic wound healing. AGEs are formed by a series of non-enzymatic reactions between saccharides, and they interact with proteins (especially long-lived proteins, such as collagen), nucleic acids, or fats [11]. Studies have reported that the deposition of AGEs in tissues is closely related to the pathological development of many diseases [12–14]. Our previous studies have shown that large amounts of AGEs deposits in skin tissue were significantly associated with wound healing [1, 8]. However, studies have not elucidated if AGEs also affect neutrophil aggregation, which ultimately leads to the dysfunction of the immune response of the skin and delayed wound healing. In the current study, we hypothesized that differences in the deposition of AGEs in different types of tissues in the skin would affect the activity and function of neutrophils in diabetic wounds and influence the attenuated formation of the inflammatory belt, which causes delayed healing of diabetic wounds.

To investigate this hypothesis, we evaluated the underlying molecular mechanisms of the abnormal migration of neutrophils in diabetic lesions, observed the characteristics of neutrophil aggregation in diabetic wound tissue, and determined the correlation between these characteristics and AGEs deposition. We also studied the effects of different levels of AGEs expression on the migratory function of neutrophils and the molecular biological mechanisms that underly this process. Overall, this study, aimed to investigate the relationship between AGEs and neutrophils in delayed diabetic wound healing in order to determine possible intervention measures.

## RESULTS

### Histological assessment of injury in diabetic rats

**Figure 1:**
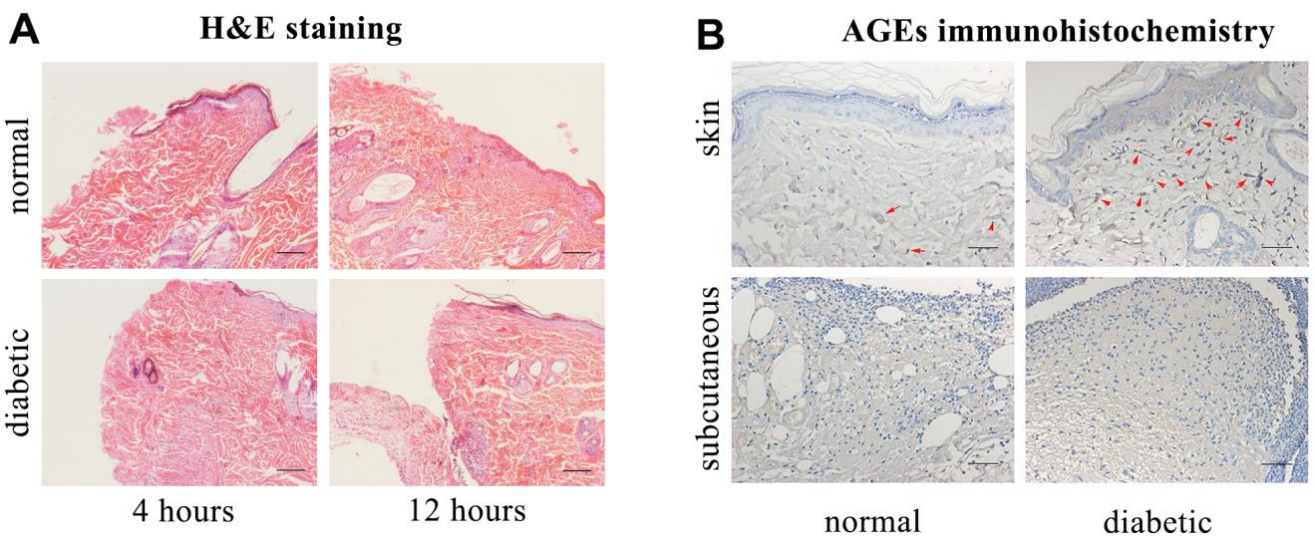
**Hematoxylin and eosin (H&E) staining, advanced glycation end products (AGEs) immunohistochemical staining.** (**A**) H&E staining. Scale bar as 0.1mm. (**B**) AGEs immunohistochemical staining (Red arrows indicate positive expressions). Scale bar as 0.025mm.

Hematoxylin and eosin (H&E) staining revealed that the inflammatory belt formed at 4 h post-injury in control rat skin tissue and at 12 h post-injury in diabetic rat skin tissue (Figure 1A).

The accumulation of AGEs was increased in the diabetic rats’ skin as compared with that in control rats’ skin, and there was no positive formation of AGEs in the diabetic or control rats’ subcutaneous edges of the wound (Figure 1B).

### AGEs and IL8RA concentrations in skin tissues with western blot

**Figure 2:**
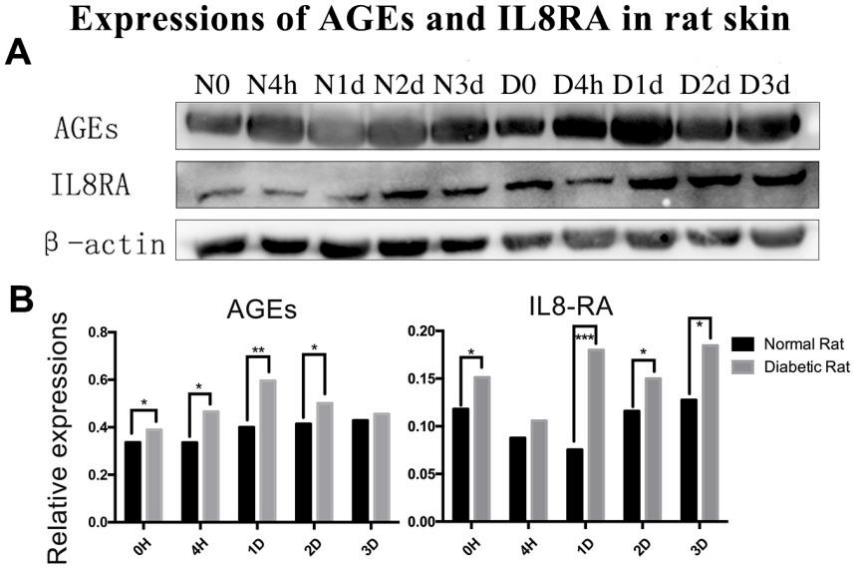
**Protein expression of advanced glycation end products (AGEs) and interleukin-8 receptor A (IL8-RA) in wound skin edge by western blotting.** (**A**) Western blotting images of advanced glycation end products (AGEs) and interleukin-8 receptor A (IL8-RA) expression. (**B**) Results of AGEs and IL8-RA expression in diabetic and control rat skin edge. *: P<0.05, **: P<0.01, ***: P<0.001. N = normal, D = diabetic, d = days, h = hours.

The level of AGEs in the diabetic rats’ skin was higher than that in the control rats’ skin before and after trauma (P<0.05, Figure 2A, 2B). The expression of interleukin-8 receptor A (IL8RA) was higher in the diabetic rats’ skin than that in the control rats’ skin, especially at 1 d post-injury (P<0.05, Figure 2A, 2B).

### The assessment of inflammatory belt

**Figure 3:**
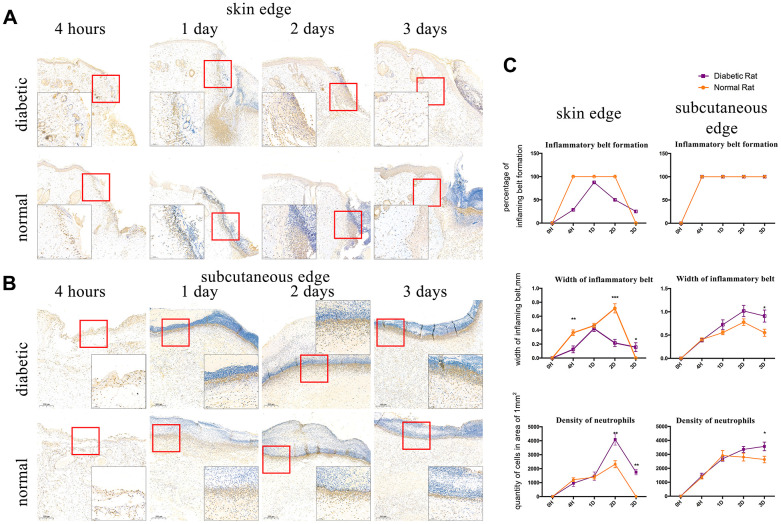
**Interleukin-8 receptor A (IL8-RA) immunohistochemical staining to determine the formation and width of the inflammatory belt and density of neutrophils.** (**A**) IL8-RA immunohistochemical staining of skin tissues. (**B**) IL8-RA immunohistochemical staining of subcutaneous tissues. (**C**) Comparation of inflammatory belt formation, inflammatory belt width, and density of neutrophils. The blue circle indicates abnormal neutrophil aggregation under the dermis of diabetic rat. *: P<0.05, **: P<0.01, ***: P<0.001.

We observed that IL8RA-positive cells formed a belt in the edge of wound, which we defined as the inflammatory belt. We evaluated the formation of the inflammatory belt at different time points after injury and calculated the proportion of the inflammatory belt that formed in the wound samples. The inflammatory belt existed in 100% (5/5) of the edges of the control rats’ skin tissue from 4 h to 2 d after injury and disappeared at 3 d post-injury (0/6, 0%). Conversely, the proportion of the inflammatory belt was compromised in the diabetic rats’ skin edge at 4 h (2/7, 28.6%), 2 d (4/8, 50%), and 3 d (2/8, 25%) after injury. The scores remained at 100% from 4 h to 3 d after injury in the edge of the subcutaneous tissues in both groups (Figure 3C).

IL8RA-positive cells with segmented nuclei were detected at high densities and identified as neutrophils. The width of the inflammatory belt that formed in the edge of skin tissue in diabetic rats at 4 h and 2 d (0.0128 ± 0.004856 mm, 0.02163 ± 0.005106 mm) after injury was lower than that in the edge of skin tissue in control rats (0.0362 ± 0.004086 mm, 0.07127 ± 0.006587 mm), and it was higher than normal at 3 d post-injury (Figure 3A, P<0.05). Conversely, the width of the inflammatory belt that formed in the edge of subcutaneous tissues in diabetic rats was higher than that in the edge of subcutaneous tissues in control rats at 1 d, 2 d, and 3 d after incision (Figure 3B, P<0.05).

The density of neutrophils was higher in the edge of skin tissues of diabetic rats (4089.725±400.44 /mm^2^, 1749.554±186.774 /mm^2^) than that in the edge of skin tissues of control rats (2317.914±254.193 /mm^2^, 0 /mm^2^) at 2 and 3 days after injury (Figure 3A, P<0.05). Additionally, the density of neutrophils in the edge of subcutaneous tissues of diabetic rats was higher (3568.392±308.108 /mm^2^) than that in the edge of subcutaneous tissues of control rats (2629.922±233.672 /mm^2^, Figure 3B P<0.05) at 3 d after injury. Interestingly, an abnormal aggregation of neutrophils also appeared at 1d after injury in the sub-dermal area around the wound of diabetic rats (Figure 3B).

### LTB4 and MPO assessment in skin tissues

**Figure 4:**
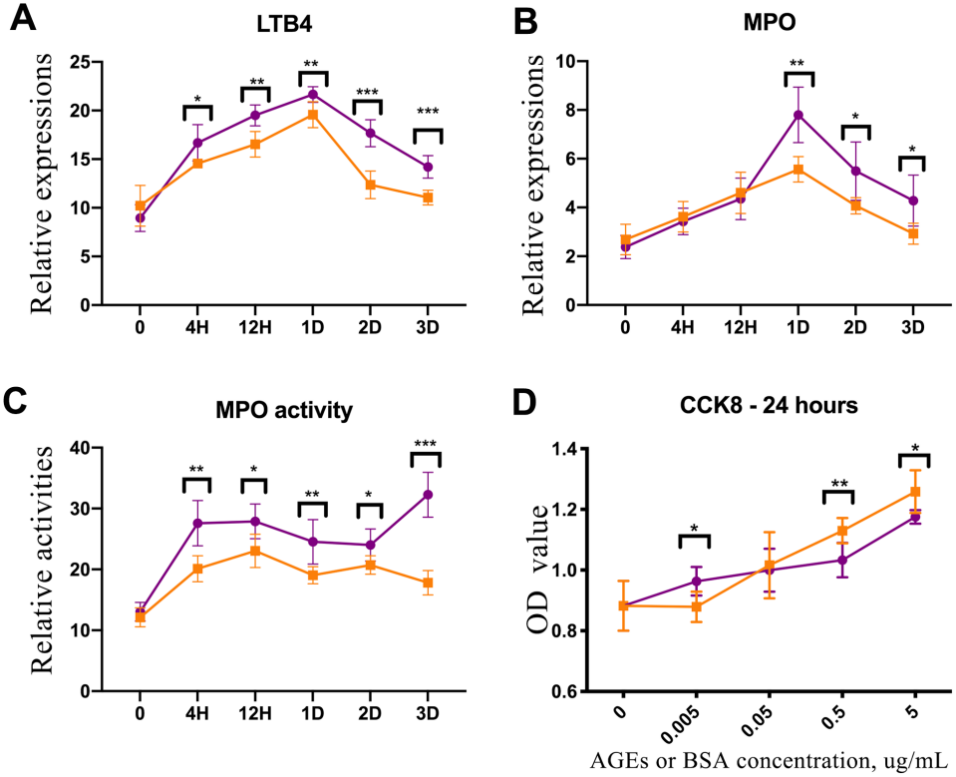
**Enzyme-linked immunosorbent assay (ELISA) experiment of rat skin and CCK-8 test of advanced glycation end product (AGE)-stimulated neutrophils.** (**A**) Leukotriene B4 (LTB4) expression in diabetic and control rat skin at different time points after injury. (**B**) Myeloperoxidase (MPO) expression in diabetic and control rat skin. (**C**) The results of MPO activity in diabetic and control rat skin. (**D**) CCK-8 test for neutrophils that were stimulated by AGEs and bovine serum albumin (BSA) for 24 h. *: P<0.05, **: P<0.01, ***: P<0.001. D = days, H = hours.

There were no differences in the concentrations of leukotriene B4 (LTB4) and myeloperoxidase (MPO) or the activity of MPO between diabetic and control rat skin tissue before injury. However, the levels of LTB4 and MPO were higher in the diabetic rats’ skin than that in the control rats’ skin after trauma (P<0.05, Figure 4A, 4C). Additionally, the concentration of MPO in the diabetic rats’ skin was elevated as compared with that in the control rats’ skin from 1 to 3 days after injury (P<0.05, Figure 4B).

### CCK-8 test of neutrophils

Neutrophils were cultured and induced using the HL-60 human cell line. AGEs were synthesized and detected *in vitro*. The activity of neutrophils that were incubated with 5 μg/ml and 0.5 μg/ml of AGEs for 24 h was down-regulated as compared with the activity of neutrophils that were incubated with 5 μg/ml and 0.5 μg/ml of BSA control. Conversely, the activity of neutrophils that were incubated with 0.005 μg/ml of AGEs was upregulated as compared with the activity of neutrophils that were incubated with 0.005 μg/ml of BSA control (P<0.05, Figure 4D). The activities of neutrophils that were stimulated with 0.05 μg/ml of AGEs and BSA were similar.

### Cell migration experiment

**Figure 5:**
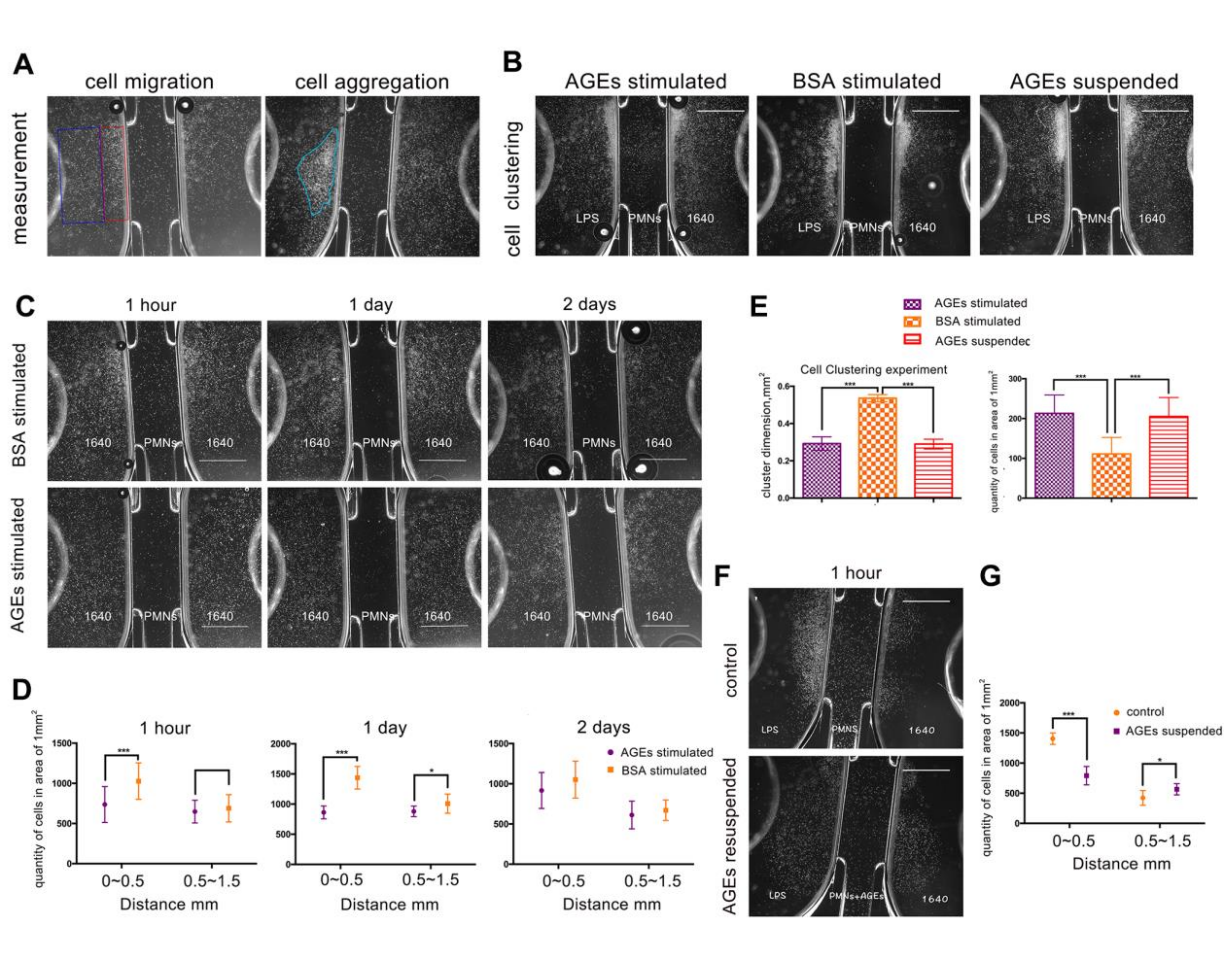
**U-slide chemotaxis experiments to observe neutrophil viability, migration, and clustering ability that was affected by advanced glycation end products (AGEs).** Scale bar as 1 mm. (**A**) Measurement of cell migration and aggregation. The blue and red squares represent middle-range and near-range areas, respectively. The blue circle represents neutrophil aggregation (clustering). (**B**, **G**) Neutrophil clustering ability that was affected by AGEs stimulation and suspension. (**C**, **E**) Neutrophil migration ability that was affected by AGEs stimulation at different time points. (**D**, **F**) Neutrophil migration ability that was affected by AGEs resuspension. *: P<0.05, **: P<0.01, ***: P<0.001.

Images from the neutrophil μ-Slide chemotaxis experiment revealed that the deposition of neutrophils that were stimulated with 0.05 μg/ml of AGEs for 24 h was more dispersed and less clustered than the deposition of neutrophil that were stimulated with 0.05 μg/ml of BSA control (Figure 5B, 5G). Additionally, the density of neutrophils was decreased in the near-range area at 1 h and 1 d after stimulation and in middle-range area at 1 day after stimulation with AGEs as compared with the density of neutrophils after stimulation with the BSA control (P<0.05, Figure 5C, 5E). The lipopolysaccharides (LPS) μ-Slide migration experiment revealed that the density of AGEs supplemented neutrophils was decreased in the near-range area and increased in the middle-range area as compared with the density of the RPMI 1640-suspended neutrophils. (P<0.05, Figure 5D, 5F). Both migratory and clustering functions were detected in the LPS migration experiment. The images from the LPS-free experiment revealed that few neutrophil clusters formed.

The dimensions of neutrophil clustering and cell density were reduced after incubation with AGEs or AGEs suspension for 24 h as compared to those after incubation with BSA for 24 h (P<0.05, Figure 5B, 5G). This finding indicated that neutrophil migration and cluster formation were reduced following AGEs stimulation as compared with those following BSA stimulation.

### RNA-sequencing and signal analysis with RT-PCR for mRNA expression

**Figure 6:**
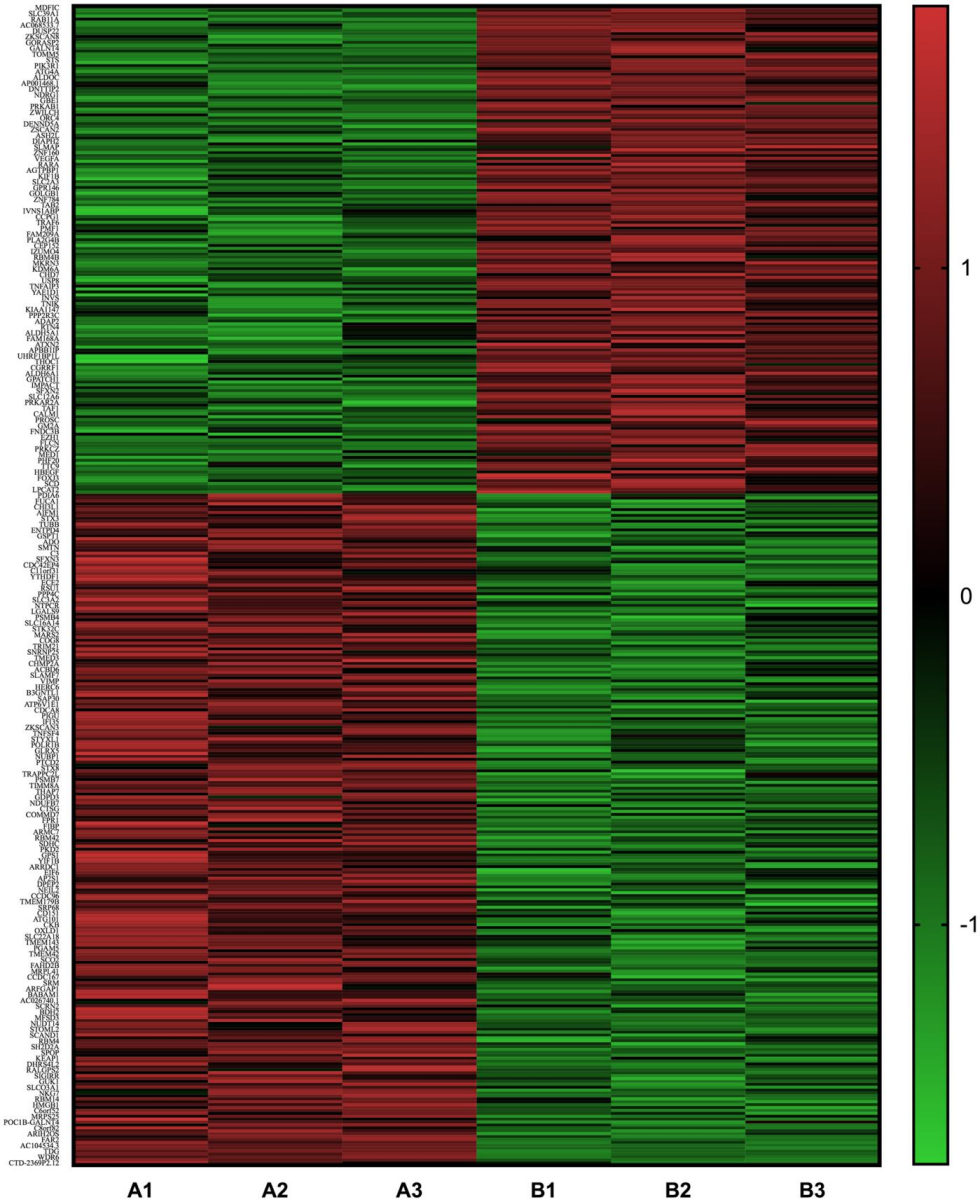
Heat map of the differentially expressed mRNA between neutrophils that were incubated with advanced glycation end products (AGEs) and neutrophils that were incubated with bovine serum albumin (BSA).

**Figure 7:**
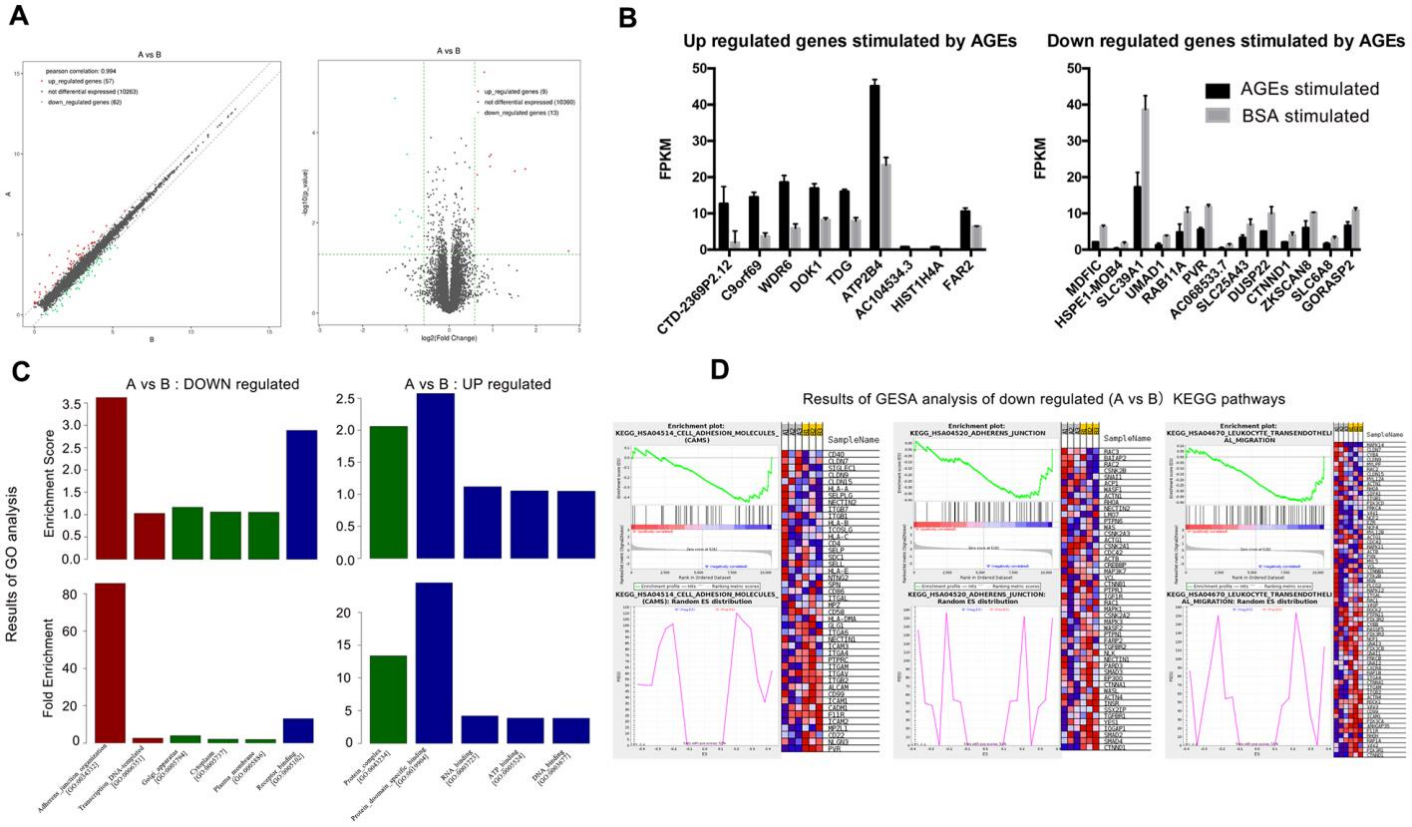
**Results of RNA-SEQ for the differential expression of mRNA between neutrophils that were incubated with advanced glycation end products (AGEs) and neutrophils that were incubated with bovine serum albumin (BSA).** (**A**) Volcano and scatter map of the differentially expressed genes between the two groups. (**B**) Significantly up- and down-regulated genes between the two groups (P < 0.05). (**C**) Results of Gene Ontology (GO) analysis. Red bars represent BP (biological process), green bars represent CC (cellular component) and blue bars represent MF (molecular function). (**D**) Results of Gene Set Enrichment Analysis (GSEA) (partial).

Significant differences (P<0.05) were detected in the mRNA expression of 399 genes between AGEs-stimulated and BSA-stimulated groups (Figure 6), and 22 genes combined with |log2FC|>0.585(FC, fold change) (Figure 7A, 7B and Table 1). Gene Ontology (GO) analysis located several genes and gene sets that changed after AGEs stimulation (Figure 7C and Table 2). Gene Set Enrichment Analysis (GSEA) revealed 98 upregulated and 164 down-regulated gene sets in the Kyoto Encyclopedia of Genes and Genomes (KEGG) pathways after stimulation with AGEs. In these gene sets, 8 were significantly upregulated and 38 were significantly down-regulated after AGEs stimulation at FDR<30% and P<0.05. Within these gene sets, 4 KEGG pathways that were related to neutrophil migration and adhesion were down-regulated (Figure 7D and Table 3). In addition, CTNND1 and PVR were significant enriched according to a GO analysis and GSEA. Raw data and detailed sequencing results can be found in the Gene Expression Omnibus (GEO) database. The series number is GES163687.

**Table 1:** 22 different expressed neutrophil mRNA between AGEs stimulated and BSA stimulated group (A vs B).

**Up or down regulation**	**Gene name**	**Fold change**	**Up or down regulation**	**Gene name**	**Fold change**
**Up regulated**	CTD-2369P2.12	6.728	**Down regulated**	MDFIC	0.419
	C9ORF69	3.370		HSPE1-MOB4	0.425
	WDR6	2.848		SLC39A1	0.454
	DOK1	1.948		UMAD1	0.455
	TDG	1.921		RAB11A	0.490
	ATP2B4	1.900		PVR	0.509
	AC104534.3	1.747		AC068533.7	0.530
	HIST1H4A	1.586		SLC25A43	0.549
	FAR2	1.564		DUSP22	0.559
				CTNND1	0.607
				ZKSCAN8	0.608
				SLC6A8	0.613
				GPRASP2	0.640

**Table 2:** Results of GO analysis (AGEs stimulated group compared with BSA group).

**Up or down regulated**	**Gene set**	**Genes**
**Up regulated**	Protein complex	ATP2B4, HIST1H4A
	Protein domain specific binding	HIST1H4A, TDG
	RNA binding	HIST1H4A, WDR6
	ATP binding	ATP2B4, TDG
	DNA binding	HIST1H4A, TDG
**Down regulated**	Adherens junction organization	CTNND1, PVR
	Transcription DNA-templated	CTNND1, MDFIC, ZKSCAN8
	Cytoplasm	CTNND1, DUSP22, HSPE1-MOB4, MDFIC, PVR
	Golgi apparatus	GORASP2, RAB11A
	Plasma membrane	CTNND1, PVR, RAB11A, SLC39A1, SLC6A8

**Table 3:** Several KEGG pathways and core genes enriched by GESA analysis that down regulated in AGEs stimulated group compared with BSA group.

**KEGG pathways**	**Core genes**
**Focal adhesion**	PIK3R1, GRB2, VEGFA, VAV2, PPP1CB, PPP1R12B, PAK4, RAP1A, BIRC2, TNR, PPP1CC, COL4A1, JUN, ARHGAP35, PIK3CA, PDPK1, VAV3, ROCK1, BRAF, SOS2, COL4A2, ACTN4, SPP1, LAMB3, ITGAV
**Cell adhesion molecules**	PVR, NLGN3, CD22, MPZL1, ICAM2, F11R, CADM1, ICAM1, CD99, ALCAM, ITGB2, ITGAV, ITGAM, PTPRC
**Adherens junction**	CTNND1, SMAD4, SMAD2, IQGAP1, YES1, TGFBR1, SSX2IP, INSR, ACTN4, WASL, CTNNA1, EP300, SMAD3, PARD3, NECTIN1
**Leukocyte transendothelial migration**	CTNND1, PIK3R1, VAV2, RAP1A, RHOH, F11R, ARHGAP35, PIK3CA, ICAM1, CD99, VAV3, ROCK1, ACTN4, ITGB2, ITGAM, CTNNA1

**Figure 8:**
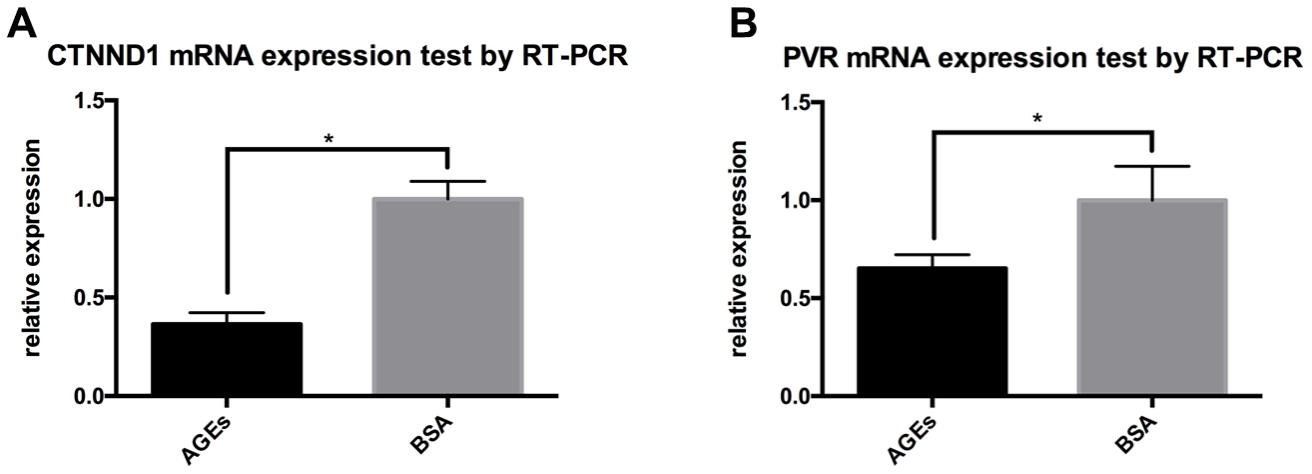
**RT-PCR results for the expression of CTNND1 and poliovirus receptor (PVR) between neutrophils that were incubated with AGEs and BSA.** *: P<0.05. (**A**) The expression of CTNND1 between the two groups. (**B**) The expression of PVR between the two groups.

Results from RT-PCR also revealed that the mRNA expressions of CTNND1 and PVR were reduced in neutrophils that were stimulated with AGEs as compared with those in neutrophils that were stimulated with BSA control (Figure 8, P<0.05).

## DISCUSSION

This study focused on the changes and mechanisms of neutrophil migration and aggregation in diabetic skin wounds. Neutrophils are essential to the innate immune response after cutaneous damage and act as an initiation factor in wound healing that leads to the accumulation and stimulation of macrophages and removal of necrotic tissues [6, 15, 16]. Therefore, it is essential for neutrophils to migrate into wound areas in a timely manner in order for the wound-healing process to occur. Diabetes mellitus and its complications, such as chronic wound, are well known and have been extensively researched by scientists [17, 18]. Most studies have focused on the differentiation and abnormal biological behavior of macrophages in the wound area, but few in-depth studies have been conducted on the activity of neutrophils [19–21]. Although some studies are limited by experimental methods, many researchers deny that neutrophils play a predominant role in wound healing. However, our team suggests that neutrophils promote wound healing and also act as one of the driving forces of macrophage chemotaxis. Moreover, the bactericidal and necrotic tissue isolation functions of neutrophils are also the first line of defense against infection [22]. The study of the biological characteristics and mechanism of neutrophils is helpful to understand the initial pattern of diabetic wound healing disorders and to provide evidence and a foundation for potential treatment options.

We established a full-thickness skin wound model in diabetic rats to investigate whether the function of neutrophils was abnormal in diabetic rats; evaluate if specific substances, such as AGEs, interfered with their biological functions, including migration, aggregation, activity, etc.; and identify the molecular mechanisms underlying these AGEs-induced changes in neutrophil function.

Although multiple studies have demonstrated that the infiltration of inflammatory cells (e.g., macrophages and neutrophils) is delayed in diabetic wounds, few studies have described the exact time points of delayed neutrophil infiltration and its profiles. We found that the formation of the inflammatory belt in diabetic rats was delayed (12 h) as compared with that in the control rats, and the width of the inflammatory belt on the edge of skin in the diabetic rats was decreased as compared with that in control rats. We initially hypothesized that there may be an obstacle preventing the aggregation and chemotaxis of neutrophils in diabetic wound. However, we observed a large number of neutrophils in the subcutaneous tissue of the wound edge of diabetic skin; therefore, this hypothesis was rejected. Studies have shown that the deficiency of neutrophils accelerated the process of wound repair in the sterile wound [23]. In our study, we found that the LTB4 protein, which is essential in neutrophil migration [15], and the activity level of MPO, which is related to the number and activity of neutrophils, in diabetic wound tissue continued to increase. Sterile wounds are difficult to locate in general, and the abnormal function and mass aggregation of neutrophils in diabetic rat skin may represent an obstacle to wound repair in this matter.

In our analysis of the time-density of neutrophils in the inflammatory belt of skin and subcutaneous tissue of diabetic rats, we found that the chemotaxis of neutrophils in subcutaneous tissue was not delayed and their aggregation ability was also enhanced. IL8RA expression showed that the total number of neutrophils in diabetic skin increased, but the formation of the inflammatory belt was delayed. We hypothesized that some substances in the dermis of diabetic rats may interfere with the formation of neutrophils into inflammatory belts. The differential deposition of AGEs in the skin and subcutaneous tissues of diabetic rats and the differential aggregation of neutrophils in these two sites may support this hypothesis. It was found that the accumulation of AGEs in the skin of diabetic rats was higher than that in skin of control rats, but there was no difference in the accumulation of AGEs in the subcutaneous tissue between diabetic rats and control rats. Therefore, it is speculated that neutrophil aggregation is closely and negatively related to the degree of AGE deposition.

AGEs interact with long-lived proteins, such as skin collagen, and are considered to be metabolic memory carriers for a long period of time; thus, the effects of AGEs on the repair of skin injury are aggravated [24]. The increased accumulation of AGEs in diabetic skin may have adverse effects on wound healing, such as increasing skin oxidative stress, reducing the scavenging of reactive oxygen species, secreting excessive TNF-α by macrophages, decreasing the proliferation and apoptosis of fibroblasts, and increasing the expression of NF-κB that inhibits the proliferation and apoptosis of keratinocytes [25–29].

In order to confirm the role of AGEs deposition in neutrophil migration and aggregation, we conducted a series of *in vitro* studies. Results from the μ-slide chemotaxis experiments confirmed that AGEs interfered with the effect of LPS on neutrophils and reduced their chemotactic (i.e., migratory) and aggregatory abilities, which were time related. AGEs can be deposited in any part of diabetic body, such as blood, local tissues, and cells. It is further speculated that the migratory and aggregatory abilities of neutrophils may be altered in diabetic patients because of their exposure to AGEs. The *in vitro* CCK-8 assay showed that low concentration of AGEs (<0.05 μg/ml) increased the activity of neutrophils, while high concentration of AGEs (>0.05 μg/ml) decreased the activity of neutrophils. This may demonstrate that AGEs are deposited at different densities that interfere with the directional recruitment and aggregation of neutrophils by LPS or other chemokines after the neutrophils are locally recruited by chemokines. As a result, a large number of neutrophils accumulated under the lower dermis of the skin.

In spite of merits, this study still has some limitations. The *in vitro* CCK-8 assay was performed on normal cell culture induced neutrophils, thus the effect of hyperglycemia and its co-effect with AGEs on neutrophils still uncovered and need further exploration. *In vitro* study of μ-Slide chemotaxis experiment illuminated neutrophil migration more visually, while this caused difficulties in analyze. To evaluate the results, we measured the coagulated area dimensions and density of neutrophils next to this area for describing the ability of migration and aggregation of neutrophils. There could be more data translated from this slide like using 3D in-visual photography.

We also investigated the molecular mechanism that may have led to these results by sequencing and analyzing the mRNA of AGEs-stimulated neutrophils and control neutrophils. After a variety of bioinformatics analyses and real-time PCR detection, PVR and CTNND1 were classified as possible candidates that caused neutrophil migration disorders in diabetes. These results provide new insights for understanding the inflammatory and immune responses in the healing process of diabetic wounds.

PVR, which encodes a transmembrane glycoprotein that belongs to immunoglobulin superfamily and connects the cell with extracellular matrix molecules in neutrophil migration, is an important adhesion protein that plays a role in the leukocyte trans-endothelial migration pathway and T cell receptor signaling pathway [28, 29]. Recent studies have indicated that PVR is related to cell adhesion and migration, adaptive immunity, and cancer [30, 31]. PVR interacts with transmembrane proteins, CD226 and TIGIT, which are essential to cell-mediated immune responses [32, 33]. Following viral infection and in cancer, DNA damage can upregulate the expression of PVR [34]. CTNND1 can be translated and expressed as p120 protein. It binds with cadherin and plays an important role in neutrophil adhesion, migration, and polarity change [35, 36]. P120 catenin regulates Rho signaling, which can directly affect the organization of the actin cytoskeleton. When released from the adherens junction, p120 promotes cell migration by regulating Rho GTPase RAC1, CDC42, and RhoA [37–39]. The loss of p120 upregulates RhoA activity and NF-κB signaling, which leads to increased MAPK signaling and dermal inflammation [40, 41]. Previous studies report that the Rho/Rho kinase signaling pathway is related to diabetic complications [42]. The down-regulation of PVR and CTNND1 mRNA in neutrophils that were stimulated by AGEs was consistent with the impairment in neutrophil migratory and adhesive functions.

Nevertheless, some cell function genes, such as TDG and ATP2B4, were upregulated following AGEs stimulation in neutrophils. These changes suggest that the migratory ability and other functions of neutrophils are affected by AGEs. It has been reported that the transcription of TDG and ATP2B4 was altered in diabetic cells or tissues [43, 44]. For this study focused on neutrophil migration and aggregation, other functions of neutrophils have not been fully illustrated and analyzed here. However, the influence of these gene variations on neutrophils remains unclear and warrants further exploration.

Nowadays, the inhibition methods of the production of AGEs have been studied and reported [45, 46]. It is still a limited area to reverse the AGEs that already formed, which will be left with expectations in the future [47, 48].

In conclusion, the results from our study suggest that the accumulation and distribution of AGEs in diabetic skin tissues compromise neutrophil migration in diabetic rat skins. Our *in vitro* studies revealed that AGEs stimulation influenced the migration and clustering of neutrophils when it reached threshold. RNA-SEQ and bioinformatics analysis confirmed that the migration and adhesion pathways were down-regulated after human neutrophils were stimulated by AGEs. Our findings suggest that the mechanism of AGEs-mediated neutrophil migration and aggregation reduction may be related to the decreased expression of CTNND1 and PVR. Further analyses of how AGEs regulate neutrophil migration with these differentially expressed genes may help us gain insights into the mechanisms underlying diabetic wound healing, guide the assessment of the severity and outcomes of diabetic wounds, and provide substantial evidence for treatment of diabetic wounds.

## MATERIALS AND METHODS

### Animals

Male Sprague Dawley rats (160-180g) were purchased from Vital River Laboratory Animal Technology (Beijing, China) and single-housed at the animal center in Ruijin Hospital in a pathogen-free environment that was maintained at 23° C under a 12-hour light/dark cycle with ad libitum access to food and water. All surgeries and procedures were performed under anesthesia (2% sodium pentobarbital; 35mg/kg) and approved by the local ethics committee.

### STZ-induced diabetic (T1D) rat model and excisional wound model

After adaptive breeding for 7 days, rats were fasted for 24 hours and randomly assigned to receive intraperitoneal injections of 50 mg/kg of streptozotocin (STZ, Sigma, USA) or citric acid buffer (control group) [49]. The OneTouch UltraVue (Johnson & Johnson, USA) was used to measure blood glucose levels weekly for 2 months (9 times). Rats that had blood glucose concentrations above 16.7 mmol/L according to all 9 OneTouch UltraVue tests were assigned to the diabetic group (n=40, 8 rats for each time point). Rats that received intraperitoneal injections of citric acid buffer and had blood glucose concentrations below 7.0 mmol/L were assigned to the control group (n=30, 6 rats for each time point).

Depilatory cream (Veet, France) was used to remove the dorsal fur 24-h before surgery. Four full-thickness (diameter: 6 mm)) excisional wounds were established using a sterile biopsy punch (Acuderm, USA). Then, the rats were sacrificed, and cutaneous wound tissue with 2~5mm wound edges was harvested s at 4 hours (h), 12 h, 1 day (d), 2 d, and 3 d post-surgery. The tissue was stored at -80° C and fixed in 10% formalin solution, respectively.

### Hematoxylin and eosin (H&E) staining

The formalin-fixed tissues were embedded into paraffin, sliced into 4-μm sections, mounted on dewaxed glass slides, and stained with hematoxylin at room temperature (RT) for 5 minutes. After staining with hematoxylin, the tissues were differentiated in 1% acid ethanol, stained with eosin for 3 minutes, dehydrated, and mounted.

### Immunohistochemical staining of IL8RA and AGEs

Interleukin-8 receptor A/chemokine receptor 1 (IL8RA/CXCR1) is primarily expressed in neutrophils, macrophages, and T cells and can be separated by the segmented morphology of the nucleus in neutrophils. Immunohistochemical staining was performed as follows. 4 μm tissues sections were sliced, dewaxed, and underwent antigen retrieval with EDTA (pH 8.0, Servicebio, China). Next, the tissues sections were incubated in 3% H_2_O_2_ for 25 min at RT and stained with an anti-IL8RA antibody (Novus, USA, 1:250, diluted in 5% BSA) or an anti-AGE antibody (Abcam, USA, 1:1000, diluted in 5% BSA) overnight at 4° C. The following day, the tissues sections were stained with an HRP-conjugated anti-rabbit secondary antibody (DAKO, Japan) for 30 min at RT, and this was followed by DAB (DAKO, Japan) staining. Next, the sections were stained with hematoxylin (Servicebio, China) for 3 min, washed, dehydrated, and mounted. Microscopic (Zeiss, Germany) images were captured using Zen digital software (Zeiss, Germany) and analyzed using Photoshop CC software.

### Evaluation of inflammatory belt

The images of the IL8RA-immunohistochemical-stained tissue slices were used to assess density of neutrophils and the width and the formation of the inflammatory belt at different time points. 5~8 slides were used in each time point group.

The percentage of inflammatory belt-formed sides was used to evaluate inflammatory belt formation in skin tissue and subcutaneous tissue. The width of each inflammatory belt (mm) was measured in 3 random positions using Photoshop CC software, and the average width of each slide was calculated. The density (mm^2^) of neutrophils in the inflammatory belts was calculated using the following equation:

density (mm^2^) = number of neutrophils (counted manually)/the area dimensions (evaluated by Photoshop CC).

### Western blotting to measure AGEs and IL8RA concentrations in diabetic and normal rat skin tissues

Tissue extracts were prepared using radioimmunoprecipitation assay (RIPA) buffer (Beyotime, China) and Protease inhibitor cocktail (Cell Signaling Technology, USA). Samples (4 replicates) underwent sodium dodecyl sulfate-polyacrylamide gel (SDS-PAGE) electrophoresis at 80 V and transferred to a polyvinylidene difluoride (PVDF) membrane (0.2 μm, Millipore, USA) at 300 mA for 90 min. Next, the membranes were blocked with 10% non-fat powdered milk (Sangon Biotech, China) for 2 h at RT and incubated overnight at 4° C with the anti-AGEs antibody (Abcam, 1:1000, USA, diluted in TBS-T) and anti-IL8RA antibody (Novus, USA, 1:500, diluted in TBS-T). The next day, the membranes were incubated with the secondary antibody for 1 hour at RT. The images were taken with the Tanon-5200 Chemiluminescent Imaging System (Tanon, China) and analyzed by Image J.

### Evaluation of LTB4, MPO, and MPO activity in skin tissues using enzyme-linked immunosorbent assay (ELISA)

Tissue extracts were prepared as described above. Enzyme-linked immunosorbent assays (ELISAs) were performed using the LTB4 ELISA kit, MPO ELISA kit, and MPO activity ELISA kit (Aoyuan Biotech, China) according to the manufacturer’s instructions.

### AGEs synthesis

AGEs were incubated *in vitro* following the processes described previously [50]. Briefly, 10g/L of bovine serum albumin (BSA) was co-incubated with 0.5mol/L D-glucose and 1.5mmol/L PMSF in sterile phosphate-buffered saline (PBS) at 37° C for 2 months. The BSA control was incubated similarly but without D-glucose. The solutions were dialyzed in sterile PBS using a dialysis bag (8000~12000 Dalton, 3M, USA), and AGEs and BSA were assessed using a fluorescence spectrometer with an excitation and emission of 370 nm and 440 nm, respectively. The concentrations of AGEs and BSA were detected using a BCA Protein Quantification Kit (Thermo Fisher, USA).

### Cell culture, differentiation, and CCK-8 test

The HL-60 cell line was provided by Dr. Yang Peilang, identified, and cultured in RPMI1640 (Gibco, USA) with 10% fetal bovine serum (FBS) (Hyclone, US). All cells were mycoplasma free and tested on June 11, 2018. Before stimulation, 1 μmol/L of all-trans-retinoid acid (ATRA; Sigma, USA) was added to the cell culture for 7 days to induce cell differentiation. DAPI (Invitrogen, USA) immunofluorescence staining was used to identify the segmented nucleus of neutrophils.

Neutrophil viability was evaluated after 24 h or 48 h following incubation with AGEs and BSA at different concentrations (5 μg/mL, 0.5 μg/mL, 0.05 μg/mL, 0.005 μg/mL, and 0 μg/mL), CCK-8 examinations were performed in replicates of 6 according to the manufacturer’s instructions (Dojindo, Japan).

### Neutrophils incubation/stimulation using AGEs or BSA

Neutrophils were cultured in RPMI1640 with 1% FBS and separated into the two following groups: group A received 24 h or 48 h of stimulation with 0.05 μg/mL of AGEs and group B received 24 h or 48 h of stimulation with 0.05 μg/mL of BSA. After stimulation, the neutrophils were prepared for the cell migration and RNA-SEQ experiments to exam the migratory abilities and changes in mRNA, respectively.

### Cell migration experiment

Cell migration (6 replicates) and clustering (3 replicates) abilities were measured using μ-Slide Chemotaxis (ibidi, Germany) according to the manufacturer’s instructions. 6 replicates represented for 6 neutrophil culture plates were used in each group to detect cell migration ability and 3 replicates represented for 3 neutrophil culture plates were used in each group to detect cell clustering ability. Approximately 3×10^6^/mL of neutrophils were used for the migration experiment, and 3×10^7^ /mL of neutrophils were used for clustering experiment. As shown in Figure 5A, 3 chambers were injected with RPIM1640, 1 μg/mL LPS that was diluted in RPIM1640), and neutrophils that were stimulated by AGEs or BSA/suspended in RPIM1640 or AGEs. Slides that were preheated in an incubator at 37° C were then mounted on the microscope stage that was sustained at a temperature of 37° C. The plugs were pulled, and the images were collected after 1 h, 1 d, or 2 d.

The results were estimated using two methods. First, we scaled the dimension of the cluster formation using Photoshop CC software. Second, we counted the density of the neutrophils in the near-range (i.e., 0~0.5 mm) and middle-range (i.e., 0.5~1.5mm) areas using Photoshop CC and Image J software.

### RNA- sequencing and bioinformatics analysis

To identify the possible molecular mechanisms that are associated with the abnormal migration of neutrophils, we performed RNA-sequencing to detect changes in mRNA expression in neutrophils after they were stimulated with AGEs and BSA control for 24 h.

Briefly, Trizol (Invitrogen, USA) was used to extract the RNA of stimulated neutrophils (3 replicates). Then, mRNA was isolated using Next Poly (A) mRNA Magnetic Isolation Module (NEB, USA), prepared using the library by KAPA Stranded RNA-Seq Library Prep Kit (Illumina, USA), and quantified using qPCR and sequencing with Illumina HiSeq 4000. All operations were performed according to the manufacturer’s instructions.

Differentially expressed genes were analyzed and enriched by GO, GSEA, and KEGG.

### RT-PCR for mRNA expression

RT-PCR was performed in replicates of 3 using the total RNA that was extracted from stimulated neutrophils as described above. RNA quantification was performed using the Applied Biosystems™ 7500 using GoTaq® 1-step RT-qPCR System (Promega, USA) according to the manufacturer’s instructions. Detailed primer sequences are described in the Supplementary Materials.

### Statistical methods

Independent-samples t-tests were used to perform statistical analyses after Levene’s Test for equality of variances, and data are presented as mean ± SD. Differences between groups were considered significant at P<0.05. The bioinformatics data were analyzed as mentioned previously.

## Supplementary Material

Supplementary Materials
