# Supplementary material for: Effects of advanced glycation end products on neutrophil migration and aggregation in diabetic wounds
Source: Aging (Albany NY). 2021 Apr 26;13(8):12143–59. doi: 10.18632/aging.202924 (PMC8109105; doi:10.18632/aging.202924)
Supplement: Supplementary Materials [file aging-13-202924-s001.pdf]

SUPPLEMENTARY MATERIALS

Primer sequences

1. GAPDH (internal reference)
   Forward: 5’ GGACCTGACCTGCCGTCTAG3’
   Reverse: 5’ GTAGCCCAGGATGCCCTTGA3’

2. CTNND1
   Forward: 5’TTGGATTCCGCTTGGATGCTGTC 3’
   Reverse: 5’CCGCACGTCAGTCTTCACCTTG 3’

3. PVR
   Forward: 5’ATGGTGAATCTGGCAGCATGGC 3’
   Reverse: 5’CGTGACGAACAGGCAGGTGTAG 3’
